# The effectiveness of non-pharmacological treatments for auditory verbal hallucinations in schizophrenia spectrum disorders: A systematic review and meta-analysis

**DOI:** 10.1192/j.eurpsy.2025.10115

**Published:** 2025-10-09

**Authors:** Melis Cobandag, Natasha Sigala

**Affiliations:** Department of Clinical Neuroscience, Brighton and Sussex Medical School, University of Sussex, Brighton, UK

**Keywords:** auditory verbal hallucinations, psychosis, schizophrenia, voices

## Abstract

**Background:**

Schizophrenia is a chronic severe mental illness affecting 24-million people globally, associated with a life expectancy 15 years shorter than the general population. Approximately 70% of people with schizophrenia experience auditory verbal hallucinations (AVHs), i.e. ‘hearing voices’. Current treatment approaches remain unsuccessful in up to 30% of cases.

**Aims:**

This systematic review and meta-analysis evaluated randomised controlled trials (RCTs) of non-pharmacological treatments for AVHs in schizophrenia spectrum disorders, assessing emerging treatment effectiveness and identifying research gaps.

**Methods:**

A literature search was performed between 2013-2024 across five databases: PubMed, Embase, PsycINFO, Medline, and Web of Science. The meta-analysis included 45 studies based on predefined criteria and bias assessment. Effect sizes (Hedge’s g) were calculated using a random effects model with 95% confidence intervals. The study followed PRISMA guidelines and was pre-registered (PROSPERO ID: CRD42024598615).

**Results:**

Our sample included 2,314 patients and fourteen interventions. The overall mean effect size was -0.298 (95% CI, [-0.470, -0.126]), representing a medium, statistically significant effect. Subgroup analyses revealed medium, statistically significant effects for both AVATAR therapy and cognitive behavioural therapy (CBT). Conversely, repetitive transcranial magnetic stimulation (rTMS) and transcranial direct current stimulation (tDCS) showed small, non-significant effects.

**Conclusions:**

AVATAR therapy has the strongest evidence for treating AVHs, highlighting the need for large-scale RCTs and integration into treatment guidelines. CBT requires methodological standardisation. Acceptance and commitment therapy shows promise but needs further high-quality RCTs. Non-invasive brain stimulation techniques require additional trials before clinical implementation.

## Introduction

Schizophrenia is a chronic and severe mental illness affecting 24 million people globally [1], accounting for approximately 1% of the population [2]. On average, people with schizophrenia have a life expectancy 15 years shorter than the general population, and a risk of death by suicide of up to 10% [3]. Although symptoms and their severity vary widely between individuals, contributing to the complexities of treatment, one frequently experienced symptom is auditory verbal hallucinations (AVHs), i.e., “hearing voices,” present in approximately 70% of people with schizophrenia [4]. AVHs are a key contributor to schizophrenia-related morbidity and mortality by reducing quality of life and elevating the risk of violence and suicide [5, 6]. Therefore, managing AVHs is critical to reduce the morbidity of this illness, with implications for potential uses in other conditions in which AVHs are a prominent symptom, such as in schizoaffective disorder or bipolar affective disorder [7].

The current first-line treatment for schizophrenia is antipsychotic medication [8], which affords the majority of patients with effective symptom control and overall improved quality of life [9], for 30% of patients, these AVHs persist despite treatment [10–12]. People with schizophrenia can often lack insight into their condition, impacting medication adherence [13], which is compounded by the potential for severe adverse effects of antipsychotics. Some serious side effects include tardive dyskinesia [14], prolonged QT syndrome [15], and agranulocytosis, which is of particular concern when using clozapine, an antipsychotic reserved for treatment-resistant schizophrenia [16].

Although antipsychotics are an essential part of treatment regimens for those with psychotic illness, the limited efficacy of antipsychotics in treating AVHs for some patients underscores a need for alternative or additional treatments, prompting a recent shift towards non-pharmacological interventions. These interventions are diverse and include talking therapies such as cognitive behavioural therapy (CBT) [17], acceptance and commitment therapy (ACT) [18], and AVATAR therapy [19], as well as neuromodulatory approaches like repetitive transcranial magnetic stimulation (rTMS) [20] and transcranial direct current stimulation (tDCS) [21]. Some studies have also investigated the role of music therapy and mindfulness-based therapies [22, 23]. While research increasingly focuses on such approaches, emerging pharmacological strategies, such as the combined use of two long-acting injectable antipsychotics in treatment-resistant cases, are also being explored to optimise outcomes in challenging cases [24]. Non-pharmacological treatments are often trialled alongside antipsychotic medications. However, there is still uncertainty surrounding the efficacy of non-pharmacological treatments, in addition to a lack of specifically tailored guidance for their implementation in clinical settings. Reasons for this may include conflicting evidence across various studies, summarised in previous systematic reviews focusing on specific types of non-pharmacological treatments [25–28]. Moreover, previous systematic reviews have focused on specific subtypes of non-pharmacological interventions for AVHs, lacking in critical evaluation and comparison between different types of non-pharmacological interventions [25–28]. Additionally, such studies have not exclusively reviewed randomised controlled trials (RCTs), which are the most methodologically robust way to test an intervention [29]. Therefore, this study seeks to provide an evaluation of recent RCTs testing non-pharmacological treatments for AVHs in SSDs. To the best of our knowledge, this is the first meta-analysis to be conducted on all non-pharmacological interventions for AVHs in SSDs.

## Aims

The specific objectives of this systematic review and meta-analysis were:

1. To evaluate the overall efficacy of non-pharmacological interventions for AVHs based on RCTs conducted in the last decade.
2. To compare the effectiveness of different types of interventions, such as CBT, rTMS and AVATAR therapy, measured by validated rating scales.
3. To assess the methodological quality and limitations of included studies.
4. To identify gaps in research and provide recommendations for future studies and clinical guidelines.

## Methods

### Search strategy, selection criteria, and quality assessment

This systematic review and meta-analysis was performed in accordance with the *Preferred reporting Items for Systematic Reviews and Meta-Analyses (PRISMA)* guidelines [30] (see Supplementary Figure S1). The protocol was preregistered on PROSPERO (ID: CRD42024598615) on 18 November 2024.

Five electronic databases were searched on 25 November 2024. Searches were performed using MeSH terms and Boolean operators, outlined in Table 1. Full search strings are provided in Supplementary Table S2. Additional search filters were applied, detailed in Table 2.

**Table 1.** Search details

Component	Search details
Databases searched	PubMed, Web of Science, EMBASE, Medline, and PsycINFO
Date of search	25 November 2024
Keywords for AVHs	“auditory hallucination*” OR “hearing voices” OR “voice-hearing” OR “auditory hallucinations” OR (“voices” AND “psychosis”)
Keywords for disorders	“Schizophrenia”[MeSH Terms] OR schizophren* OR “psychotic disorders” OR “psychosis” OR “schizoaffective disorder”
Keywords for treatments	(“Treatment Outcome”[MeSH Terms] OR “therapy” OR “treatment efficacy” OR “antipsychotic agents”[MeSH Terms] OR “Clozapine” OR “Haloperidol” OR “Olanzapine” OR “Risperidone” OR “Aripiprazole” OR “CBT” OR “Cognitive Behavioral Therapy” OR “cognitive remediation therapy” OR “psychoeducation” OR “family therapy” OR “supportive therapy” OR “electroconvulsive therapy” OR “rTMS” OR “transcranial magnetic stimulation” OR “tDCS” OR “transcranial direct current stimulation” OR “Deep Brain Stimulation” OR “DBS” OR “virtual reality therapy” OR “VR therapy” OR “avatar therapy” OR “digital intervention” OR “mHealth” OR “eHealth” OR “psychedelic-assisted psychotherapy” OR “psilocybin”).

**Table 2.** Search filters applied to databases, and number of studies yielded

Database	No. initial search results	Additional filters	No. of results with additional filters
PubMed	930	Randomised controlled trial Human English	150
Web Of Science	1228	English Add “controlled trial” to search in all fields Publication type: Article	208
PsycINFO	1539	Clinical trial English Peer reviewed Adult	75
Medline	1249	Humans English Randomised controlled trial	138
EMBASE	4811	Human English language Randomised controlled trial Publication type: Article Source type: Journal	146
Total:	9757		717

Inclusion criteria for studies were a) Adults aged ≥18 years, b) the majority of participants with a formal diagnosis of schizophrenia or other psychotic disorder, c) RCTs, d) any treatment/therapy for auditory hallucinations, e) papers published in English, f) papers published from 2013 and onwards. This publication date cutoff was used to focus on the most recent and clinically relevant studies, avoid overlap with earlier reviews, and maintain a manageable scope given the growing body of literature. Papers including participants with substance misuse or a coexisting neurological condition were excluded. All other study types that were not RCTs were excluded.

The *Standard Quality Assessment Criteria for Evaluating Primary Research Papers from a Variety of Fields*, also known as the *QualSyst Tool* [31] detailed in Table 3, was used to evaluate the risk of bias of each article that met the inclusion criteria. Risk of bias scores for each study were calculated by both reviewers independently, then compared to evaluate inter-operator agreement, and an inclusion threshold of 0.55 was used. Bias assessment was conducted on all 77 papers, which underwent full-text screening for eligibility. All papers met the 0.55 threshold; therefore, no studies were excluded based on methodological quality. The final selection of 45 studies included in the meta-analysis was based on the previously detailed inclusion criteria, including the availability of AVH-specific outcome data. If an article met all eligibility criteria but lacked data on the AVH measure, corresponding authors were contacted. Out of 14 corresponding authors contacted, 2 responded with the post-treatment data required [23, 32]. The other 11 studies were not included in this meta-analysis [33–43]. Another paper was excluded [44] due to the author responding to explain that their paper reanalysed the data reported in study 31. *QualSyst* bias assessment scores for each study are provided in Supplementary Table S3.

**Table 3.** QualSyst risk of bias assessment process

Each question was scored using the following:	Questions used to assess risk of bias:
	Question/objective sufficiently described?
Yes = 2	Study design evident and appropriate?
Partially = 1 No = 0	Method of subject/comparison group selection or source of information/input variables described and appropriate?
N/A = exclusion of specific question in overall assessment	Subject (and comparison group, if applicable) characteristics sufficiently described?
	If interventional and random allocation was possible, was it described?
Scores are reported as a proportion of the total, where a perfect score of 28 points is represented as 1.00	If interventional and blinding of investigators was possible, was it reported?
	If interventional and blinding of subjects was possible, was it reported?
Final Score 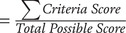	Outcome and (if applicable) exposure measure(s) well defined and robust to measurement/misclassification bias? Means of assessment reported?
	Sample size appropriate?
	Analytic methods described/justified and appropriate?
	Some estimate of variance is reported for the main results?
	Controlled for confounding?
	Results reported in sufficient detail?
	Conclusions supported by the results?

### Data synthesis and analysis

Data extraction started on 17 December 2024. The following information was extracted from each article: setting, patient sex, age, ethnicity, diagnosis, other treatments participants received (other than the intervention of interest), intervention tested, control condition, frequency and dosage of intervention, AVH scoring system, number of participants in each group, mean and standard deviations of the AVH score at baseline and post-treatment, and a summary of key findings.

The primary outcome measure was the difference in post-treatment AVH scores between the control group and intervention group, where the AVH score measured at least one of the following: frequency, distress, or intensity of AVH. The data required to calculate change scores and standard deviations were inconsistently reported; therefore, to avoid making assumptions, using post-treatment AVH scores was most suitable. This is appropriate in the absence of change score data, given that there are minimal baseline differences between the groups. Baseline scores were carefully considered for all studies, and were similar across groups, providing a suitable and comparable measure of treatment effect.

Analyses were performed using the software *Comprehensive Meta-Analysis Version 4 (CMA V4)*, [45] which assessed the heterogeneity of the studies, and calculated the effect size for each study and overall. Forrest plots were produced, using a random effects model with 95% confidence intervals. The heterogeneity of the studies (the outcome variability due to clinical and methodological differences) was measured with Higgins’s I^2^ statistic [46]. Higgins’s I^2^ represents the percentage of variation between the sample estimates that is due to heterogeneity rather than sampling error [46]. Subgroup analyses were performed for an intervention reported in at least five studies.

Risk of publication bias was independently assessed by both reviewers through visual inspection of the funnel plot, which was created using *CMA V4* [47].

## Results

**Figure 1.**
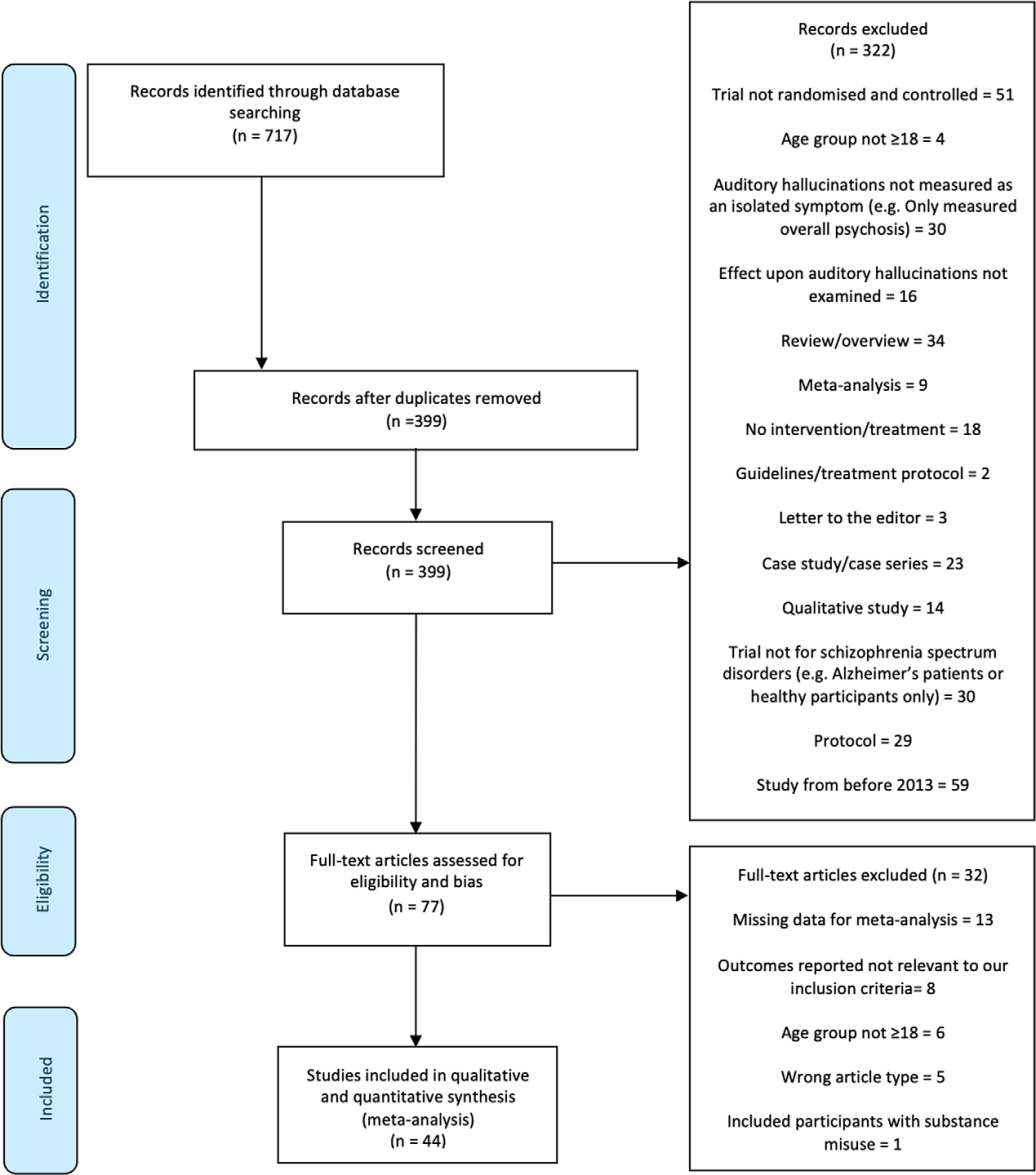
*PRISMA* flow diagram for study inclusion.

The initial search produced 717 records. After removal of duplicates, titles, and abstracts of 399 articles were screened. If titles and abstracts appeared relevant, full texts were retrieved. 77 full-text articles were assessed for eligibility. 45 articles met all eligibility criteria and were included in the meta-analysis (Figure 1).

**Figure 2.**
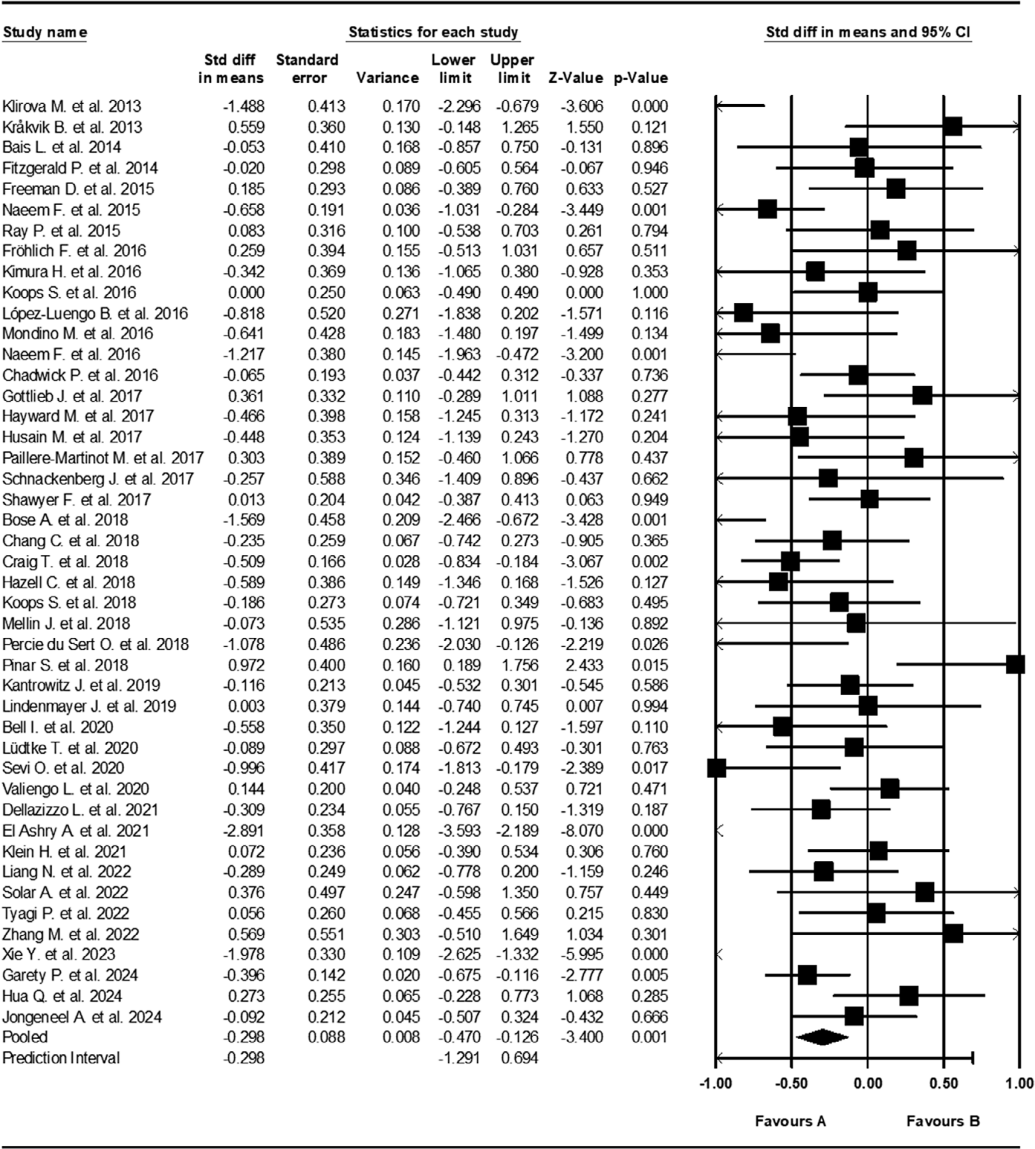
Forest plot of standardized mean differences (SMDs) between post-intervention AVH scores in those who received an intervention for AVHs (A), versus the control group (B). Negative effect sizes indicate lower (improved) AVH scores in the intervention group compared to the control group.

### Study characteristics and participant characteristics

Studies identified included the following 14 types of treatment: rTMS, CBT, transcranial direct current stimulation (tDCS), AVATAR therapy, transcranial alternating current stimulation (tACS), smartphone app treatments, acceptance and commitment therapy (ACT), psychological online intervention (POI), relating therapy (RT), counselling, music therapy (MT), referral to a community psychologist, group mindfulness-based intervention, and rehabilitation. Supplementary Figure S1 reports the number of each intervention type included in the analyses.

For all studies, sample sizes ranged from 12 to 201 participants, with a median of 37. Inclusion criteria across studies involved adults diagnosed with schizophrenia or related disorders who experienced AVHs. Various AVH scoring systems were used across studies, including the *Auditory Hallucinations Rating Scale (AHRS)* [48], *Positive and Negative Syndrome Scale – Auditory Hallucinations (PANSS AH)* [49], *Psychotic Syndrome Rating Scales – Auditory Hallucinations (PSYRATS AH)* [48], *The Hamilton Program for Schizophrenia Voices Questionnaire (HPSVQ)* [50], *Delusion and Voices Self-Assessment (DV-SA)* [51], and *Characteristics of Auditory Hallucination Questionnaire (CAHQ)* [52–54]. Each scoring system included a measurement of at least one of frequency, distress, or intensity of AVHs. For all AVH scoring systems used, a lower score indicated better AVH outcomes. A change in baseline to post-treatment scores was interpreted as reflecting treatment effects, with a decrease in scores indicating improvement.

Trials were conducted across 20 different countries. The majority of studies (31) were conducted in Western countries, with an emerging representation (12) from Asia. There was an underrepresentation from Africa and Latin America, with only two studies conducted in these regions.

In all but two trials [55, 56], it was stated that the intervention being explored was administered alongside usual treatments, with a common theme of maintaining stable medication regimes. This approach ensured that any observed changes could be attributed to the intervention, rather than changes in established routine treatments.

Participant demographics and study characteristics are detailed in Tables 4 and 5, respectively.

**Table 4.** Demographics of included studies [17–23, 32, 55–91]

Study	Diagnosis	Mean age (SD)	Sex (% male)	Treatments participants were maintained on
Klirova et al. [57]	Paranoid schizophrenia	35.9 (9.5) t	60.0	Antipsychotics, antidepressants, anticonvulsants, anxiolytics
Kråkvik et al. [58]	Schizophrenia, delusional disorder, or schizoaffective disorder	35.3 (8.9) i 37.5 (11.2) c	64.4	Medication decided by psychiatrist
Bais et al. [59]	Schizophrenia	37.2 (14.9) i 37.3 (11.6) c	59.4	Antipsychotics
Fitzgerald et al. [55]	Schizophrenia or schizoaffective disorder	39.3 (11.7) t	62.5	Not specified
Freeman et al. [60]	Schizophrenia	39.6 (11.6) i 42.2 (13.5) c	68.2	Antipsychotics, hypnotics, anxiolytics, antidepressants medication
Naeem et al. [61]	Schizophrenia or related disorders based on ICD–10 criteria	59.0 (31.7) i 57.0 (31.1) c	61.3	TAU including antipsychotic medications and nursing care.
Ray et al. [62]	Schizophrenia	31.4 (7.1) i 29.3 (8.7) c	Not provided	Psychotropic medication
Fröhlich et al. [21]	Schizophrenia, Schizoaffective disorder	43.4 (12.6) i 40.0 (10.7) c	84.6	Antipsychotics, benzodiazepines, anticonvulsants
Kimura et al. [63]	Schizophrenia	44.6 (10.5) i 40.7 (9.0) c	46.7	Antipsychotics
Koops et al. [64]	Schizophrenia, schizoaffective disorder, schizophreniform disorder, psychosis not otherwise specified.	38.0 (15.0) i 42.0 (13.0) c	56.3	Antipsychotics
López-Luengo et al. [65]	Schizophrenia, schizoaffective disorder, schizophreniform disorder.	34.0 (11.6) i 29.3 (7.7) c	81.3	Antipsychotics
Mondino et al. [66]	Schizophrenia	36.7 (9.7) i 37.3 (9.7) c	65.2	Antipsychotics
Naeem et al. [67]	Schizophrenia or related psychotic disorders.	42.0 (11.5) i 38.6 (12.0) c	52.0	TAU (not specified)
Chadwick et al. [23]	Schizophrenia, Schizoaffective disorder	Not provided	50.0	TAU, including 2–3 monthly outpatient appointments, antipsychotic medication, contact with care team every two weeks.
Gottlieb et al. [68]	Schizophrenia, schizoaffective disorder, or psychosis not otherwise specified.	43.8 (13.2) i 40.3 (11.7) c	62.2	TAU, including regular clinical services and stable pharmacological treatment.
Hayward et al. [69]	Schizophrenia spectrum disorder (62.1%), borderline personality disorder, depression.	Not provided	55.0	TAU, including antipsychotic medication, psychiatric outpatient appointments, and regular contact with clinical care teams.
Husain et al. [70]	Schizophrenia, schizoaffective disorder, delusional disorder.	34.1 (9.6) i 30.5 (8.2) c	67.1	TAU (not specified)
Paillere-Martinot et al. [71]	Schizophrenia, schizoaffective disorder	32.1 (6.8) i 31.3 (7.8) c	55.1	Antipsychotics, antidepressants, benzodiazepines, and mood stabilisers
Schnackenberg et al. [72]	Schizophrenia, schizoaffective disorder	44.1 (9.5) i 40.2 (11.3) c	58.3	TAU, including antipsychotic medication, general rehabilitative life skills support, and techniques for the distraction from voices or challenging beliefs.
Shawyer et al. [18]	Schizophrenia, schizoaffective disorder	35.6 (15.3) i 33.0 (8.5) c	61.5	Antipsychotics
Bose et al. [73]	Schizophrenia	31.3 (8.3) i 31.4 (7.6) c	55.8	Antipsychotics
Chang et al. [74]	Schizophrenia	46.4 (10.3) i 42.2 (10.3) c	45.0	Antipsychotics
Craig et al. [75]	Paranoid schizophrenia (77%), schizoaffective disorder (11%), unspecified psychosis (5%), bipolar disorder, depression with psychotic symptoms.	40.5 (10.1) i 42.9 (11.2) c	68.0	Antipsychotics
Hazell et al. [76]	Psychosis spectrum disorders (46.3%), borderline personality disorder, mood disorder.	35.3 (8.3) i 39.6 (11.4) c	39.3	Antipsychotics
Koops et al. [77]	Schizophrenia	44.0 (11.0) i 44.0 (12.0) c	46.3	Antipsychotics
Mellin et al. [78]	Schizophrenia	29.6 (11.0) i 38.9 (10.0) c	Not provided	Antipsychotics
Percie du Sert et al. [56]	Schizophrenia	42.9 (12.4) i 42.9 (12.4) c	66.7	Not specified
Pinar et al. [22]	Schizophrenia	37.0 (10.7) i 32.8 (7.9) c	21.4	Antipsychotics
Kantrowitz et al. [79]	Schizophrenia	38.2 (9.9) i 40.1 (8.6) c	75.1	Antidepressants, anticholinergic/extrapyramidal symptoms treatments, mood stabilisers, anxiolytics, sleep aids and stimulants.
Lindenmayer et al. [80]	Schizophrenia, schizoaffective disorder	40.2 (10.7) t	85.7	Antipsychotics
Bell et al. [81]	Schizophrenia (38.3%), schizoaffective disorder (38.3%), schizophreniform (3.0%), unspecified schizophrenia spectrum disorder (3.0%), bipolar disorder with psychotic features, or major depression with psychotic features.	39.1 (10.7) i 42.6 (10.6) c	44.1	TAU comprising of standard care provided by a clinical team, including antipsychotic medication and case management.
Lüdtke et al. [82]	Non-affective psychotic disorder	41.7 (9.9) i 41.4 (9.3) c	43.6	Antipsychotics, psychotherapeutic treatment, and psychiatric consultations.
Sevi et al. [17]	Schizophrenia	36.8 (9.1) t	74.4	Antipsychotics
Valiengo et al. [83]	Schizophrenia	35.3 (9.3) t	80.0	Benzodiazepines, antipsychotics.
Dellazizzo . et al. [84]	Schizophrenia, schizoaffective disorder	43.6 (12.0) i 41.4 (13.4) c	75.7	TAU, including no change in existing medication.
El Ashry et al. [85]	Schizophrenia	27.7 (2.8) t	100.0	Antipsychotics
Klein et al. [86]	Schizophrenia spectrum disorders	41.2 (10.5) i 41.2 (10.5) c	55.1	Antipsychotics
Liang et al. [87]	Schizophrenia	25.3 (5.3) i 26.5 (6.8) c	47.5	Medications unchanged.
Solar et al. [88]	Schizophrenia	Not provided	Not provided	Referral opportunity to a community psychologist (intervention
Tyagi et al. [89]	Schizophrenia	32.2 (11.2) i 33.3 (11.5) c	64.4	Antipsychotics
Zhang et al. [90]	Schizophrenia	37.0 (13) i 45.0 (10) c	64.0	Antipsychotics
Xie et al. [32]	Schizophrenia	30.3 (4.5) i 31.5 (6.4) c	Not provided	Antipsychotics
Garety et al. [19]	Schizophrenia (43.8%), schizoaffective disorder (7.8%) persistent delusional disorder (0.9%), acute and transient psychotic disorders (1.2%), induced delusional disorder, non-organic psychotic disorders, unspecified non-organic psychosis, bipolar affective disorder, or severe depressive episode with psychotic symptoms.	40.8 (13.7) i 38.7 (12.8) c	61.4	TAU typically involving antipsychotic medication, contact with a mental health worker, and outpatient psychiatric appointments.
Hua et al. [20]	Schizophrenia	26.9 (7.2) i 27.8 (9.4) c	46.8	Antipsychotics
Jongeneel et al. [91]	Schizophrenia (43.8%), psychotic disorder not otherwise specified (20.2%), schizoaffective disorder (1.1%), post-traumatic stress disorder, borderline personality disorder, or mood disorder.	44.7 (12.3) i 41.3 (10.3) c	44.8	Antipsychotics

*Abbreviations:* c, control group, I, intervention group, TAU, treatment as usual, t, total combined value for both the intervention and control groups.

**Table 5.** Characteristics of included studies [17–23, 32, 55–91]

Study	Intervention	Control	Geographical location	Setting (inpatient or outpatient)	Sample size	AVH scoring system
Klirova et al. [57]	rTMS	Sham rTMS	Czech Republic	Not specified	15i 15c	AHRS
Kråkvik et al. [58]	CBT	TAU	Norway	Not specified	23i 22c	PSYRATS AH physical
Bais et al. [59]	rTMS	Sham rTMS	The Netherlands	In and out	16i 16c	AHRS
Fitzgerald et al. [55]	tDCS	Sham tDCS	Canada	Not specified	13i 11c	PANSS AH
Freeman et al. [60]	CBT	TAU	UK	Not specified	22i 25c	PSYRATS Hallucinations
Naeem et al. [61]	CBT	TAU	Pakistan	Not specified	59i 57c	PSYRATS AH
Ray et al. [62]	rTMS	Sham rTMS	India	Not specified	20i 20c	AHRS
Fröhlich et al. [21]	tDCS	Sham tDCS	USA	Not specified	13i 13c	AHRS
Kimura et al. [63]	rTMS	Sham rTMS	Japan	Not specified	16i 14c	AHRS
Koops et al. [64]	rTMS	Sham rTMS	The Netherlands	Not specified	32i 32c	AHRS
López-Luengo et al. [65]	Rehab (attention training programme)	No intervention	Spain	Not specified	8i 8c	PSYRATS AH
Mondino et al. [66]	tDCS	Sham tDCS	France	Not specified	11i 12c	AHRS
Naeem et al. [67]	CBT	TAU	Canada	Not specified	18i 15c	PSYRATS AH
Chadwick et al. [23]	Group mindfulness-based intervention	TAU	UK	Out	54i 54c	PSYRATS AH distress
Gottlieb et al. [68]	CBT	TAU	USA	Out	19i 18c	PSYRATS AH
Hayward et al. [69]	RT	TAU	UK	Not specified	13i 13c	PSYRATS AHRS
Husain et al. [70]	CBT	TAU	Pakistan	Out	17i 16c	PSYRATS hallucinations severity
Paillere-Martinot et al. [71]	rTMS	Sham rTMS	France	Out	15i 12c	AHRS
Schnackenberg et al. [72]	Counselling	TAU	Germany	Out	7i 5c	PSYRATS Voices
Shawyer et al. [18]	ACT	Befriending	Australia	Not specified	49i 47c	PSYRATS AH distress (frequency included as a covariate)
Bose et al. [73]	tDCS	Sham tDCS	India	Out	12i 13c	AHRS
Chang et al. [74]	tDCS	Sham tDCS	Taiwan	Not specified	30i 30c	AHRS
Craig et al. [75]	AVATAR therapy	Supportive counselling	UK	Not specified	75i 75c	PSYRATS AH
Hazell et al. [76]	CBT	Waitlist control receiving TAU	UK	Out	14i 14c	HPSVQ
Koops et al. [77]	tDCS	Sham tDCS	The Netherlands	Not specified	28i 26c	AHRS
Mellin et al. [78]	tDCS	Sham tDCS	USA	Not specified	7i 7c	AHRS
Percie du Sert et al. [56]	AVATAR therapy	TAU	Canada	Out	15i 7c	PSYRATS AH
Pinar et al. [22]	MT	No intervention	Turkey	In	14i 14c	CAHQ
Kantrowitz et al. [79]	tDCS	Sham tDCS	USA	In and out	47i 42c	AHRS
Lindenmayer et al. [80]	tDCS	Sham tDCS	USA	In	15i 13c	AHRS
Bell et al. [81]	Smartphone app	TAU	Australia	Not specified	17i 17c	PSYRATS AH
Lüdtke et al. [82]	POI	Waitlist control with no active intervention	Switzerland	Out	16i 39c	DV-SA
Sevi et al. [17]	CBT	Routine care	Turkey	Out	12i 14c	PSYRATS AH
Valiengo et al. [83]	tDCS	Sham tDCS	Brazil	Out	50i 50c	AHRS
Dellazizzo et al. [84]	AVATAR therapy	CBT	Canada	Not specified	37i 37c	PSYRATS AH
El Ashry et al. [85]	ACT	TAU	Egypt	In	35i 29c	PSYRATS AH
Klein et al. [86]	tDCS	Sham tDCS	USA and Ireland	Not specified	36i 36c	AHRS
Liang et al. [87]	AVATAR therapy	CBT	China	Not specified	32i 33c	PSYRATS AH
Solar et al. [88]	Referral to community psychologist	TAU	Australia	In	7i 10c	PSYRATS AH
Tyagi et al. [89]	cTBS	Sham tDCS	India	In and out	30i 29c	PSYRATS AH
Zhang et al. [90]	tACS	Sham tACS	USA	Not specified	14i 11c	AHRS
Xie et al. [32]	rTMS	Placebo tDCS	China	Not specified	30i 25c	AHRS
Garety et al. 2024 [19]	AVATAR therapy	TAU	UK	Not specified	98i 103c	PSYRATS AH
Hua et al. [20]	rTMS	Sham rTMS	China	Not specified	32i 30c	AHRS
Jongeneel et al. [91]	Smartphone app	AVH monitoring on smartphone app	The Netherlands	Out	44i 45c	AHRS

*Abbreviations:* ACT, acceptance and commitment therapy; AHRS, Auditory Hallucinations Rating Scale; c, control group; CAHQ, Characteristics of Auditory Hallucination Questionnaire; CBT, cognitive behavioural therapy.; DV-SA, Delusion and Voices Self-Assessment; HPSVQ, The Hamilton Program for Schizophrenia Voices Questionnaire; i, intervention group; MT, music therapy; PANSS AH, Positive and Negative Syndrome Scale – Auditory Hallucinations; PSYRATS AH, Psychotic Syndrome Rating Scales – Auditory Hallucinations; RT, relating therapy; rTMS, repetitive transcranial magnetic stimulation; tACS, transcranial alternating current stimulation; tDCS, transcranial direct current stimulation.

### Synthesis of results

#### Overall

**Figure 3.**
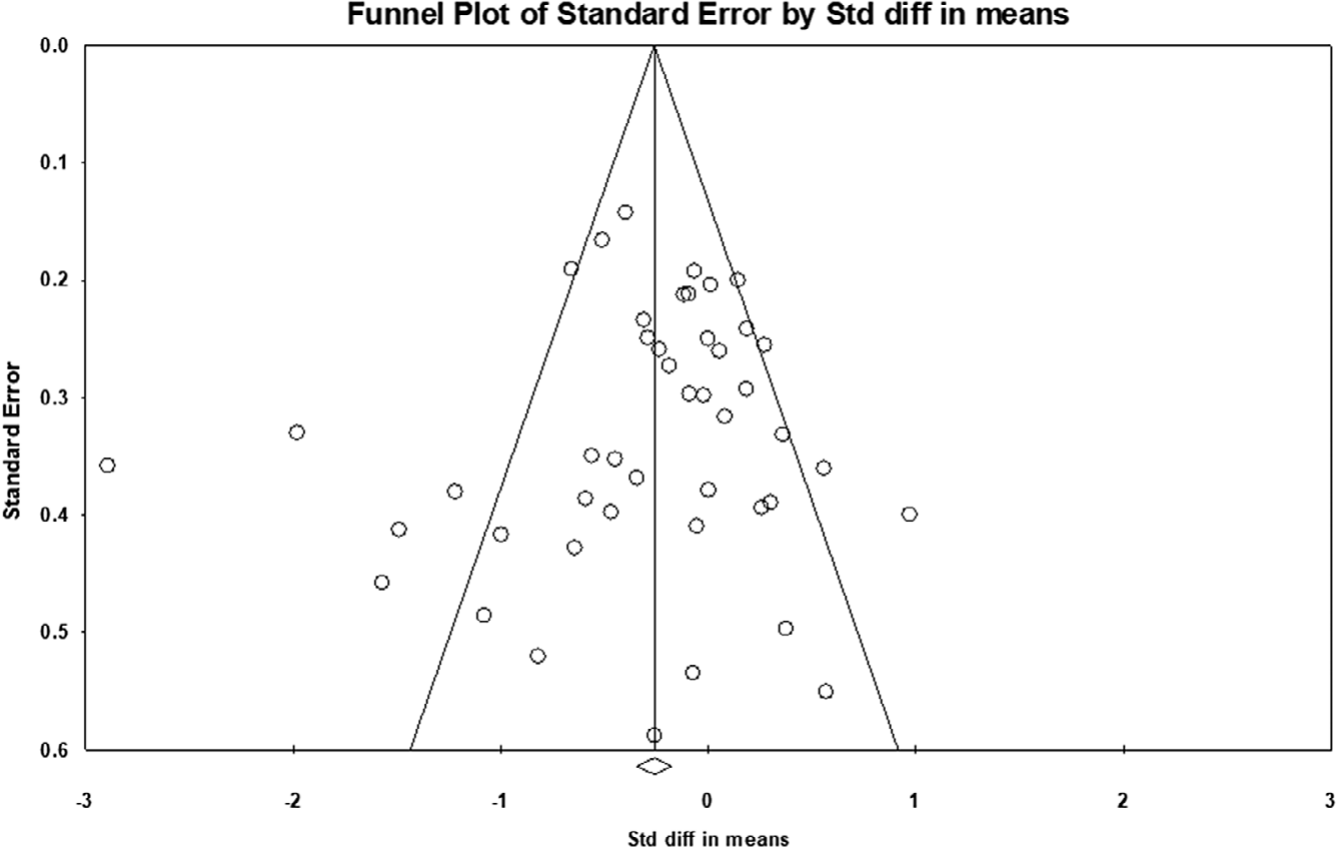
Funnel plot for publication bias.

Our sample size included 2,314 participants across all studies. The funnel plot (Figure 3) revealed an even distribution of studies reporting both positive and negative effects, and no bias for studies reporting larger, rather than smaller, effect sizes. An additional precision funnel plot was created to provide a clearer depiction of the relationship between study size and effect size (Supplementary Figure S2). Overall, a medium and statistically significant effect size of −0.298 (95% CI, [−0.470, −0.126], *Z* = −3.400, *p* = 0.001) was observed across all non-pharmacological interventions (Figure 2). There was significant heterogeneity between studies (*Q* = 171.566 with 44 degrees of freedom, *p* < 0.001, *I* ^2^ = 74%), indicating substantial variability in effect sizes.

### Subgroup analysis of interventions

As significant heterogeneity was determined, subgroup analyses of AVATAR therapy, CBT, rTMS, and tDCS were performed to explore whether different intervention types influenced effect sizes.

### AVATAR therapy

**Figure 4.**
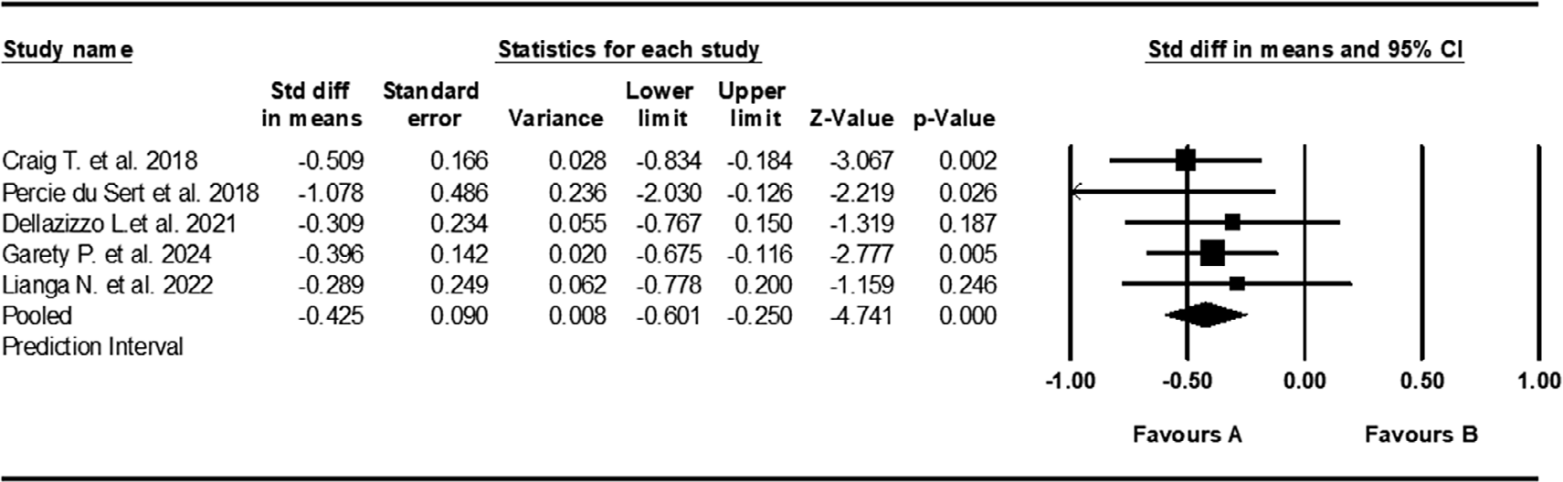
Forest plot of standardized mean differences (SMD) between post-intervention AVH scores in those who received AVATAR therapy for AVHs (A), versus the control group (B). Negative effect sizes indicate lower AVH scores in the intervention group compared to the control group.

Five studies tested the use of AVATAR therapy and had sample sizes ranging from 22 to 201 with a median of 74 [19, 56, 75, 84, 87]. All studies used the *PSYRATS AH* scoring system and were carried out across three different countries and continents: the UK, Canada, and China. AVATAR therapy showed a medium and statistically significant effect size of −0.425 (95% CI, [−0.601, −0.250], *Z* = −4.741, *p* < 0.001) (Figure 4). Effect sizes were highly consistent across studies, showing no significant heterogeneity (*Q* = 2.649, *I* ^2^ = 0%).

### Cognitive behavioural therapy

**Figure 5.**
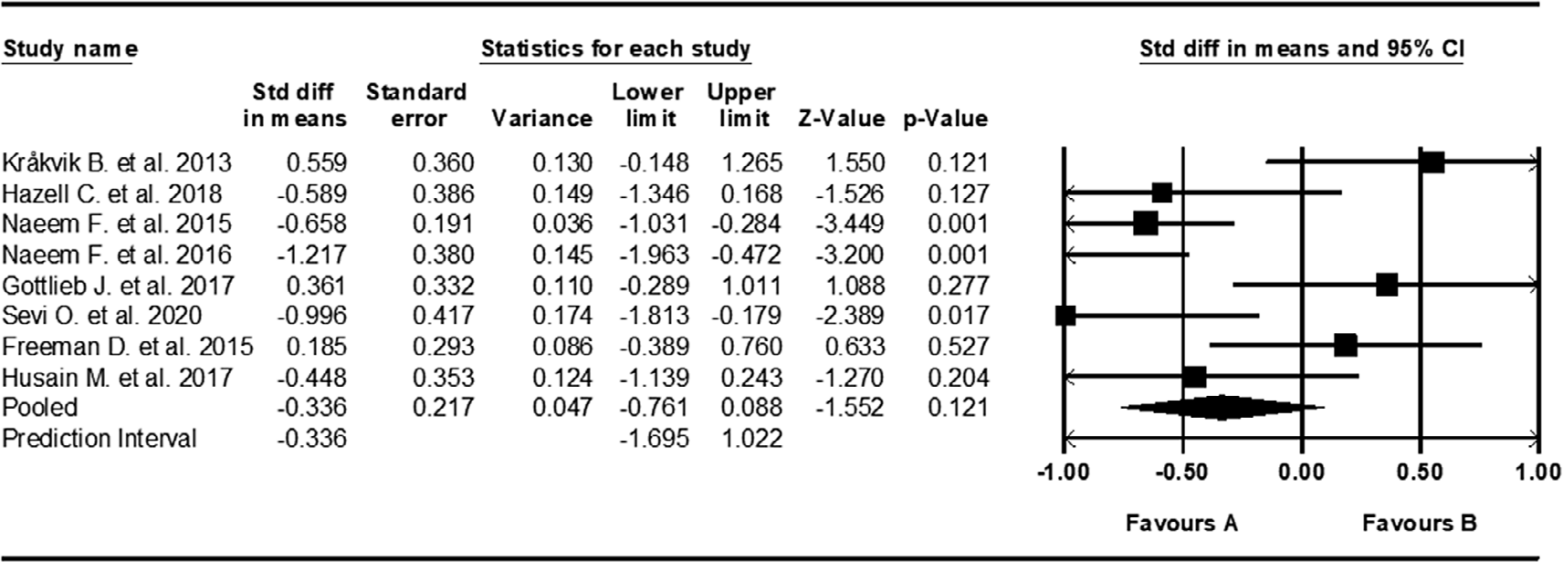
Forest plot of standardized mean differences (SMD) between post-intervention AVH scores in those who received CBT for AVHs, versus the control group. Negative effect sizes indicate lower AVH scores in the intervention group compared to the control group.

Eight studies tested the use of CBT and had sample sizes ranging from 26 to 116, with a median of 35 [17, 58–61, 67–70, 76]. AVH scoring systems varied between studies, including both *HPSVQ* and *PSYRATS AH.* Studies were carried out across six different countries including Pakistan, the UK, Canada, the USA, Norway, and Turkey. CBT showed a medium and statistically significant effect size of −0.396 (95% CI, [−0.764, −0.027], *Z* = -2.103, *p* = 0.035) (Figure 5). Effect sizes were highly inconsistent across studies, showing moderate and statistically significant heterogeneity (*Q* = 19.624, *I* ^2^ = 64%, *p* = 0.006).

### Repetitive transcranial magnetic stimulation

**Figure 6.**
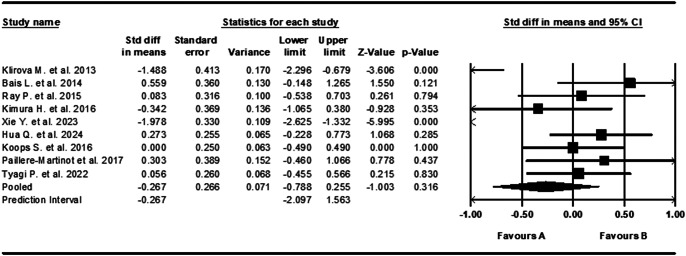
Forest plot of standardized mean differences (SMD) between post-intervention AVH scores in those who received rTMS for AVHs (A), versus the control group (B). Negative effect sizes indicate lower AVH scores in the intervention group compared to the control group.

Nine studies tested the use of rTMS and had sample sizes ranging from 27 to 64, with a median of 36 [20, 32, 57, 59, 62–64, 71, 89]. All studies used the AHRS and were carried out across six different countries: the Czech Republic, the Netherlands, France, India, Japan, and China. rTMS showed a small and not statistically significant effect size of −0.267 (95% CI, [−0.788 to 0.255], *Z* = −1.003, *p* = 0.316) (Figure 6). Effect sizes were highly inconsistent across studies, showing substantial and statistically significant heterogeneity (*Q* = 50.842, *I* ^2^ = 84%, *p* < 0.001).

### tDCS

**Figure 7.**
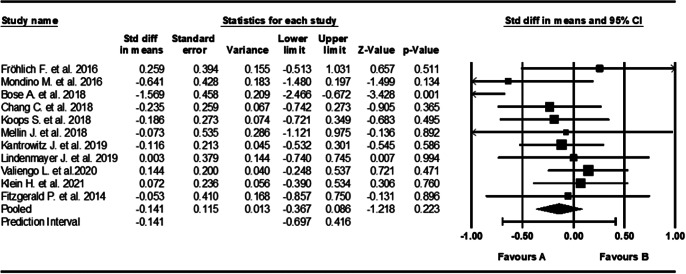
Forest plot of standardized mean differences (SMD) between post-intervention AVH scores in those who received tDCS for AVHs (A), versus the control group (B). Negative effect sizes indicate lower AVH scores in the intervention group compared to the control group.

Eleven studies tested the use of tDCS and had sample sizes ranging from 7 to 50, with a median of 13 [21, 55, 66, 73, 74, 77–80, 83, 86]. Scoring systems used varied between studies, including both the *AHRS* and *PANSS AH.* Studies were carried out across eight different countries, including: USA, France, India, Taiwan, the Netherlands, Brazil, Ireland, and Canada. tDCS showed a very small and not statistically significant effect size of −0.141(95% CI, [−0.367, 0.086], *Z* = −1.085, *p* = 0.278) (Figure 7). Effect sizes were inconsistent across studies, showing moderate, but not statistically significant heterogeneity (*Q* = 16.105, *I* ^2^ = 34%, *p* = 0.097).

### Transcranial alternating current stimulation

The study by Zhang et al. [90] reported a medium-sized, non-significant effect, favouring those in the control group (*Hedge’s g* = 0.569 (95% CI, [−0.510, 1.649], p = 0.301, Z = 1.034).

### Smartphone app

Two studies trialled a smartphone app intervention. The study by Bell et al. [81] trialled blended coping-focused therapy using ecological momentary assessment and intervention via a smartphone app. They found a medium, but not statistically significant effect size favouring the intervention (*Hedge’s g* = −0.558, 95% CI [−1.22, 0.127], *p* = 0.11, *z* = -1.597). The study by Jongeneel et al. [91] trialled the use of the smartphone app, *Temstem*, which consists of two language games: “Silencing” to improve control over voices, and “Challenging” to reduce vividness and emotionality of voices. No significant difference was observed between those using the two language games on the *Temstem* app, and those in the control group (*Hedge’s g* = −0.092, 95% CI [−0.507, 0.324], *p* = 0.666, *z* = −0.432).

### Psychological online intervention

Lüdtke et al. [82] found that partaking in a POI resulted in no significant difference in AVHs compared to the control group (Hedge’s *g* = −0.089, 95% CI [−0.672, 0.493], *p* = 0.763, *z* = −0.301).

### Acceptance and commitment therapy

Two studies trialled ACT. *Shawyer et al.* [18] found that ACT demonstrated no significant difference in AVHs compared to the control group (*Hedge’s g* = 0.013, 95% CI [−3.87, 0.413], *p* = 0.949, *z* = 0.063). In contrast, El Ashry et al. [85] found that ACT demonstrated a very large and statistically significant effect size, favouring the intervention (*Hedge’s g* = −2.891, 95% CI [−3.595, −2.189], *p* < 0.001, *z* = −8.07).

### Music therapy

Rast tonality MT, explored by Pinar et al., [22] found a large, statistically significant effect size favouring the control group, who did not listen to any music whilst in hospital (*Hedge’s g* = 0.972, 95% CI [0.189, 1.756], *p* = 0.015, *z* = 2.433).

### Relating therapy

Hayward et al. [69] found that RT showed a medium, but not statistically significant effect size, favouring the intervention (*Hedge’s g* = −0.466, 95% CI [−1.245, 0.313], *p* = 0.241, *z* = −1.172).

### Counselling

*Schnackenberg et al.* [72] found that experienced focused counselling, had a very small and non-significant effect size, favouring the intervention (*Hedge’s g* = −0.092, 95% CI [−0.507, 0.896], *p* = 0.662, *z* = 0.437).

### Rehabilitation (attention training programme)

The study by López-Luengo et al. [65] found that rehabilitation in the form of an attention training programme produced a large, but not statistically significant effect size, favouring the intervention (*Hedge’s g* = −0.818, 95% CI [−3.593, −2.189], *p* = 0.116, *z* = −1.571).

### Referral to community psychologist

Solar et al. [88] found that referring participants to a community psychologist resulted in a medium and non-significant effect size, favouring the control, which involved TAU (standard hospital care, with no direct psychologist appointment, but could self-request a referral) (*Hedge’s g* = 0.376, 95% CI [−0.598, 1.350], *p* = 0.449, *z* = 0.757).

### Group mindfulness-based intervention

Chadwick et al. [23] found that receiving a group mindfulness-based intervention resulted in no significant difference in AVHs compared to the control group (Hedge’s *g* = −0.065, 95% CI [−0.442, 0.312], *p* = 0.736, *z* = −0.337).

## Discussion

### Summary of findings

This systematic review and meta-analysis summarised evidence from the last decade on fourteen different non-pharmacological interventions for schizophrenia and related disorders, through an evaluation of RCT studies. AVATAR therapy demonstrated the most favourable results for treating AVHs and was highly consistent across all studies. The larger sample sizes and geographical spread of RCTs exploring AVATAR therapy increase the generalisability of results. Overall, AVATAR therapy stands out as a promising candidate for future clinical implementation for treating AVHs; however, further replication across diverse settings and populations is needed before widespread adoption. CBT had promising, and statistically significant results of moderate effect size for improving AVHs; however, substantial heterogeneity was observed between studies. This may be due to the differences in methodologies employed in different trials, such as differences in CBT techniques, treatment duration, and therapist expertise. Non-invasive brain stimulation techniques, including rTMS, tDCS, and tACS did not yield statistically significant results in this meta-analysis; however, this may reflect variabilities in protocols, insufficient power, or other study-level factors rather than a definitive lack of clinical effect. However, rTMS had large variability in results across different studies, suggesting that differences in methodologies, potentially concerning anatomical location, and exact rTMS protocol, along with possible differences in patient populations, may be impacting the results. In contrast tDCS studies showed no significant heterogeneity, with more studies consistently reporting non-significant results, suggesting that either tDCS may not be clinically effective for AVHs, or that current studies lack sufficient power or methodological consistency to detect an effect. This conclusion is reinforced by the fact that 11 studies, conducted across the globe, were included in the subgroup analysis, increasing the diversity of samples and, therefore generalisability of the results. ACT demonstrated variable effectiveness at treating AVHs across the two studies. However, despite limited evidence for ACT, the study by El Ashry A. et al. [85] had a moderate sample size, a very high *QualSyst* bias assessment score suggestive of low risk of bias, and was the only study carried out in Africa, a continent frequently underrepresented in research. As therapy is culturally influenced [92], demonstrating this effect in a non-Western setting could have great implications for effectively treating AVHs in these contexts. However, the contrasting results found by Shawyer et al. [18] demonstrate a need for more trials refining the most effective method for delivering ACT. Rehabilitation, counselling, RT, and referral to a community psychologist all showed improvement in AVHs post-treatment, but these changes did not reach statistical significance, potentially due to limited power, sample size, or variability in study design. Receiving a group mindfulness-based intervention did not show statistical improvement in reducing AVHs. Similarly, smartphone apps and POI also did not yield statistically significant improvements in AVHs, potentially reflecting delivery challenges, suggesting that online platforms may benefit from future refinements before they can be considered for clinical applications. Lastly, MT demonstrated significantly better AVH outcomes in the control group post-treatment, who did not listen to any music during their hospital stay. However, at follow up, there was an improvement in AVHs in those who received MT, highlighting the value of long-term and follow-up RCTs for the treatment of AVHs.

### Future research directions and clinical implications

By comparing these findings to existing systematic reviews and meta-analyses, we can assess the consistency, divergence, and potential advancements in understanding the efficacy of treatments for AVHs in schizophrenia spectrum disorders. A previous systematic review by Dellazizzo et al. [25] explored relational-based therapies for AVHs, including both AVATAR therapy and CBT. Whilst CBT focuses on changing the person’s cognition surrounding their AVHs by, for instance, altering their belief that they must obey their AVHs, AVATAR therapy works on improving the relationship between the person and their AVHs, aiming to give the patient a sense of control. The present meta-analysis found that AVATAR therapy had a moderate size and statistically significant effect favouring the intervention. Dellazizzo et al.’s reported large or very large effect sizes for AVATAR therapy. The difference in effect size may be partially explained by the fact that the previous review included studies ranging from very low to high risk of bias, whereas only studies with low risk of bias were included in the present review. The reliability of our findings is reinforced by the low heterogeneity for AVATAR therapy (*I* ^2^ = 0.00%), making the conclusion valuable for future evidence-based clinical practice. However, it is important to note that three of the five included studies were conducted in the UK, with one in Canada and one in China, suggesting a need for greater geographic and cultural diversity in future trials. Continued research is needed to replicate these findings across more diverse populations and healthcare contexts before widespread clinical adoption can be recommended. Future RCTs should therefore not only evaluate AVATAR therapy’s efficacy but also assess its adaptability across different cultural settings to inform its potential integration into clinical practice.

According to the *National Institute for Health and Care Excellence (NICE)*, CBT is currently a recognised treatment for schizophrenia and psychosis and is included in the *NICE* guidelines as a best-practice treatment recommendation [93]. However, evidence from Dellazizzo et al.’s [25] systematic review reports that across the five studies included, all but one case series failed to show any significant effects of CBT in reducing PSYRATS AH scores. Similarly, the high heterogeneity across the eight studies included in this meta-analysis (*I* ^2^ = 64%) warrants cautious interpretation of the moderate and statistically significant effect size found in favour of CBT. This prompts an exploration of the different methodologies and samples used in trials for CBT. More high-quality research following the methodology of successful trials is needed to increase the reliability of results and to refine the most effective way to deliver CBT. Given the heterogeneity of the findings and mixed statistical significance, further targeted research is needed to clarify for whom and under what conditions CBT is most clinically effective for AVHs. Therefore, guidelines should be revised to specify which patient groups may benefit from CBT, as it is currently a general recommendation for those with schizophrenia or psychosis [93]. However, if AVHs are a main, distressing, and debilitating symptom for an individual, CBT may not be the appropriate treatment for them, and guidelines should reflect symptomology rather than general diagnoses, particularly in the context of schizophrenia spectrum disorders. Additionally, alternative therapies should be considered for best-practice guidelines, such as AVATAR therapy which has shown greater evidence for effectiveness against AVHs compared to CBT.

ACT is another variant of CBT, which focuses on helping individuals to reduce the distress caused by voices. A systematic review by Yıldız [94] included three studies which explored AVH outcomes post ACT. The study found that two out of the three studies reported significant improvements in AVHs after receiving ACT. This variability in effectiveness is also present in our results. Given the limited number of RCTs for ACT, this intervention remains under explored and future trials must be conducted to draw reliable conclusions.

With regards to non-invasive brain stimulation techniques, a systematic review and meta-analysis of ten studies by Otani et al. [26], exploring the use of rTMS for treatment of auditory hallucinations in refractory schizophrenia, found a moderate, statistically significant effect size favouring rTMS (*Hedge’s g* = 0.49, 95% CI [0.11, 0.88], p = 0.011). Conversely, we found a small, not statistically significant effect size (*Hedge’s g* = −0.267, [95% CI, −0.788, 0.255], *p* = 0.316), in favour of rTMS. Multiple factors may have contributed to this difference. First, it is important to note that there was no crossover in the papers included in this meta-analysis and *Otani et al.’s* meta-analysis, which focused on papers from the years 2000 to 2011. Additionally, *Otani et al.* included studies that used low-frequency rTMS and rTMS applied only on the left temporoparietal cortex (leTPC) [26]. Studies in the current meta-analysis included rTMS applied at any anatomical location, including the leTPC, left temporo-parietal junction (TPJ), right TPJ, and personalised localisation of the language perception area. Additionally, our study included high-frequency stimulation, continuous theta-burst stimulation, and low frequency stimulation. These differences may indicate that low-frequency rTMS applied exclusively to the leTPC may be more effective in treating AVHs, highlighting a need for more trials exploring this, and an up-to-date meta-analysis on studies with this inclusion criterion. However, the meta-analysis by Otani et al. did not evaluate each paper for risk of bias prior to inclusion, which may have also affected the results.

Furthermore, a previous systematic review and meta-analysis of 16 studies exploring tDCS by Cheng et al. [27] found a moderate and statistically significant effect size in favour of tDCS (SMD = 0.36, 95 % CI [0.02, 0.70]). Although tDCS is also a non-invasive brain stimulation technique, it utilises an electrical current applied across the patient’s scalp [95], versus the use of pulsed magnetic fields in rTMS [96]. tDCS also has greater potential for use in clinical practice due to being more cost-effective and easily accessible than rTMS [97]. However, Cheng et al. observed substantial heterogeneity across the included studies (*I* ^2^ = 65%), and statistical significance did not survive subsequent sensitivity analyses. Similarly, our meta-analysis found a small and not statistically significant effect size in favour of tDCS (−0.141, 95% CI, [−0.367 to 0.086]), with no significant heterogeneity, reinforcing the reliability of the negative result. Another systematic review of nine studies exploring tDCS by Rashidi et al. found that tDCS was no better than sham tDCS in reducing AVH in 3 studies, and that tDCS had a significant effect in reducing AVHs in the remaining six studies [28]. The variability in results across systematic reviews suggests that more high-quality RCTs exploring tDCS may be needed to confirm effectiveness.

### Strengths and limitations

This systematic review and meta-analysis has a number of strengths. First, the inclusion of a large sample of 45 studies with a total of 2,314 participants increases the statistical power of the meta-analysis and provides a comprehensive summary of evidence from the last decade. Second, PRISMA guidelines were followed, and a robust bias assessment was carried out on each included study, along with an assessment of a funnel plot for publication bias. The funnel plot did not demonstrate obvious publication bias, suggesting that a range of studies with both positive and negative findings were published [98]. This increases the reliability of our results. Since only high-quality evidence was included, results can be used to influence future research priorities, with the potential to influence clinical practice and healthcare policy. Furthermore, some subgroup analyses had low heterogeneity, particularly for AVATAR therapy. This enables a definite interpretation of effect size [46], which can provide a clear guide for future research plans and clinical applications. The strengths of the present study make it a valuable addition to the literature, including previous scoping reviews on non-pharmacological treatments of AVHs [99].

This systematic review and meta-analysis also has some limitations, which must be addressed. First, 11 eligible studies had to be excluded for missing data regarding pre- and post-treatment AVH scores, despite contacting authors with data requests. Missing data can introduce bias, as negative or non-significant results may have been omitted, leading to a potential overestimation of effect sizes [100]. Second, while some studies reported follow-up outcomes, this study did not. Consequently, it was not possible to assess the long-term treatment effects; an essential consideration for clinical applicability, as therapeutic impacts may wane, persist, or intensify over time [101]. The absence of long-term data limits the strength of conclusions regarding sustained efficacy and patient outcomes. Future RCTs should incorporate follow-up assessments, and meta-analyses would benefit from stratifying and rigorously evaluating longitudinal data. It is important to acknowledge that, due to inconsistent reporting of change scores and associated standard deviations across studies, treatment effects were derived from post-treatment AVH scores. While baseline AVH scores were similar between groups in the included studies, using post-treatment scores rather than change scores may reduce sensitivity to within-group effects and should be considered when interpreting the results. Additionally, there was variability and inconsistency in the assessment tools used to measure AVHs across studies. While all included studies measured at least one core domain of AVHs, such as frequency, intensity, or distress, they employed different psychometric instruments and scales. This variability introduces measurement heterogeneity, which may have complicated data synthesis and interpretation. To account for this, standardised effect sizes were used in the meta-analysis; however, caution is warranted when comparing across studies due to differences in measurement approaches. Furthermore, some subgroups showed substantial and significant heterogeneity in their results, particularly for rTMS and CBT. This makes findings harder to interpret, as high heterogeneity may weaken the generalisability and reliability of pooled effect sizes. For example, findings may reflect study specific factors, rather than true treatment efficacy [102]. Importantly, lack of statistical significance in some findings should not be interpreted as evidence of no clinical effect. Many interventions may show real clinical benefit that is not detectable due to small sample sizes, methodological limitations, or inadequate statistical power. Future high-quality studies are needed to clarify these effects.

Furthermore, all included studies evaluated the interventions as adjuncts to existing treatment regimens, making it impossible to isolate their specific effects on AVHs. This limitation compromises internal validity, but may enhance external validity by better reflecting how such interventions are implemented in real-world clinical and community settings [103]. The inclusion of some participants who did not have SSDs, but instead had mood or personality disorders with AVHs, may limit how reliably conclusions can be drawn about the treatment of AVHs in SSDs specifically. Future meta-analyses should adopt rigid inclusion criteria for diagnoses. Nonetheless, the present meta-analysis included such studies to ensure a comprehensive overview of all recent RCTs for treatments of AVHs, where the majority of participants had SSDs. Additionally, studies support that AVHs may be phenomenologically similar across these conditions [104, 105] and respond similarly to established treatments such as antipsychotics [104]. Furthermore, not all studies had a placebo, inactive, or TAU control group. For instance, the study exploring AVATAR therapy by Liang et al. [87] utilised an active control group which received CBT, on top of TAU. This makes the true effect of the intervention unclear, as the two interventions were compared head-to-head.

Despite incorporating a wide range of keywords related to emerging non-pharmacological treatments, such as virtual reality therapy, digital interventions (eHealth, mHealth), and psychedelic-assisted psychotherapy, few or no eligible RCTs were identified for many of these approaches. This underscores the need for rigorous, well-powered RCTs to evaluate the efficacy of these novel interventions for AVHs. Addressing these underrepresented domains through targeted investigation may enrich evidence-informed practice and enhance the diversity of therapeutic avenues available to mental health professionals.

## Conclusion

This systematic review and meta-analysis highlight that AVATAR therapy has the strongest base of evidence for treating AVHs in SSDs. It is of clinical priority to continue large-scale RCTs for AVATAR therapy, including studies across culturally and geographically diverse populations, to confirm its effectiveness and generalisability. Conversely, CBT requires standardisation to improve reliability with regards to its effectiveness in treating AVHs. This finding supports the ongoing dialogue around the refinement of clinical guidelines, such as those proposed by NICE, towards more symptom-oriented recommendations for SSDs. Although ACT has shown potential in alleviating AVH-related distress, its application in clinical settings warrants additional empirical validation and methodological refinement. Moreover, there remains a clear need for geographically diverse RCTs, including the expansion of studies like El Ashry et al.’s [85] that examine ACT in non-Western populations. Finally, while non-invasive brain stimulation techniques appear promising, current evidence is insufficient to justify their integration into clinical practice.

## Supporting information

10.1192/j.eurpsy.2025.10115.sm001 Cobandag and Sigala supplementary material Cobandag and Sigala supplementary material

## Data Availability

Data availability is not applicable to this article as no new data were created or analysed in this study.

## Supplementary material

The supplementary material for this article can be found at http://doi.org/10.1192/j.eurpsy.2025.10115.
