# Supplementary material for: The effectiveness of non-pharmacological treatments for auditory verbal hallucinations in schizophrenia spectrum disorders: A systematic review and meta-analysis
Source: Eur Psychiatry. 2025 Oct 9;68(1):e155. doi: 10.1192/j.eurpsy.2025.10115 (PMC12646125; doi:10.1192/j.eurpsy.2025.10115)
Supplement: Cobandag and Sigala supplementary material [file S0924933825101156sup001.docx]

**Supplementary materials**

1. PRISMA-P (or equivalent) table
2. Search strings used for various platforms such as MEDLINE, Scopus etc.
3. Number of each intervention type included
4. List of all included papers
5. Publication bias: funnel plot
6. Quality Assessment

**Table S1.** PRISMA 2020 checklist: Key items to report in a systematic review.

| **Section and Topic** | **Item #** | **Checklist item** | **Location where item is reported** |
| --- | --- | --- | --- |
| **TITLE** | | |  |
| Title | 1 | Identify the report as a systematic review. | Page 1 |
| **ABSTRACT** | | |  |
| Abstract | 2 | See the PRISMA 2020 for Abstracts checklist. | Page 2 |
| **INTRODUCTION** | | |  |
| Rationale | 3 | Describe the rationale for the review in the context of existing knowledge. | Pages 3-4. Introduction, paragraphs 3-4. |
| Objectives | 4 | Provide an explicit statement of the objective(s) or question(s) the review addresses. | Page 4. Aims section. |
| **METHODS** | | |  |
| Eligibility criteria | 5 | Specify the inclusion and exclusion criteria for the review and how studies were grouped for the syntheses. | Page 6. Methods, Paragraph 3 |
| Information sources | 6 | Specify all databases, registers, websites, organisations, reference lists and other sources searched or consulted to identify studies. Specify the date when each source was last searched or consulted. | Page 5-6, Methods, Tables 1 and 2. |
| Search strategy | 7 | Present the full search strategies for all databases, registers and websites, including any filters and limits used. | Page 6, Methods, Table 2 and supplementary Table S2. |
| Selection process | 8 | Specify the methods used to decide whether a study met the inclusion criteria of the review, including how many reviewers screened each record and each report retrieved, whether they worked independently, and if applicable, details of automation tools used in the process. | Page 6-7, Methods, Paragraph 4. |
| Data collection process | 9 | Specify the methods used to collect data from reports, including how many reviewers collected data from each report, whether they worked independently, any processes for obtaining or confirming data from study investigators, and if applicable, details of automation tools used in the process. | Methods, page 7, Data synthesis and analysis. |
| Data items | 10a | List and define all outcomes for which data were sought. Specify whether all results that were compatible with each outcome domain in each study were sought (e.g. for all measures, time points, analyses), and if not, the methods used to decide which results to collect. | Methods, page 7, Data synthesis and analysis subheading. |
|  | 10b | List and define all other variables for which data were sought (e.g. participant and intervention characteristics, funding sources). Describe any assumptions made about any missing or unclear information. | Methods, page 7, Data synthesis and analysis subheading. |
| Study risk of bias assessment | 11 | Specify the methods used to assess risk of bias in the included studies, including details of the tool(s) used, how many reviewers assessed each study and whether they worked independently, and if applicable, details of automation tools used in the process. | Page 6-7, Methods, Paragraph 4 and supplementary materials Table S3 and S4. |
| Effect measures | 12 | Specify for each outcome the effect measure(s) (e.g. risk ratio, mean difference) used in the synthesis or presentation of results. | Methods, page 7, Data synthesis and analysis subheading. |
| Synthesis methods | 13a | Describe the processes used to decide which studies were eligible for each synthesis (e.g. tabulating the study intervention characteristics and comparing against the planned groups for each synthesis (item #5)). |  |
|  | 13b | Describe any methods required to prepare the data for presentation or synthesis, such as handling of missing summary statistics, or data conversions. |  |
|  | 13c | Describe any methods used to tabulate or visually display results of individual studies and syntheses. | Methods, page 7, Data synthesis and analysis subheading. |
|  | 13d | Describe any methods used to synthesize results and provide a rationale for the choice(s). If meta-analysis was performed, describe the model(s), method(s) to identify the presence and extent of statistical heterogeneity, and software package(s) used. | Methods, page 7, Data synthesis and analysis subheading. |
|  | 13e | Describe any methods used to explore possible causes of heterogeneity among study results (e.g. subgroup analysis, meta-regression). | Methods, page 7, Data synthesis and analysis subheading. |
|  | 13f | Describe any sensitivity analyses conducted to assess robustness of the synthesized results. | Methods, page 7, Data synthesis and analysis subheading. |
| Reporting bias assessment | 14 | Describe any methods used to assess risk of bias due to missing results in a synthesis (arising from reporting biases). | Methods, page 7, Data synthesis and analysis subheading. |
| Certainty assessment | 15 | Describe any methods used to assess certainty (or confidence) in the body of evidence for an outcome. | Methods, page 7, Data synthesis and analysis subheading. |
| **RESULTS** | | |  |
| Study selection | 16a | Describe the results of the search and selection process, from the number of records identified in the search to the number of studies included in the review, ideally using a flow diagram. | Results, page 8. |
|  | 16b | Cite studies that might appear to meet the inclusion criteria, but which were excluded, and explain why they were excluded. | Methods, page 7, paragraph 4. |
| Study characteristics | 17 | Cite each included study and present its characteristics. | Page 10-15, Table 2 and Table 3. |
| Risk of bias in studies | 18 | Present assessments of risk of bias for each included study. | Supplementary materials, figure S4. |
| Results of individual studies | 19 | For all outcomes, present, for each study: (a) summary statistics for each group (where appropriate) and (b) an effect estimate and its precision (e.g. confidence/credible interval), ideally using structured tables or plots. | Results, page 16, Figure 2. |
| Results of syntheses | 20a | For each synthesis, briefly summarise the characteristics and risk of bias among contributing studies. | Results, page 16-17, paragraph 1. |
|  | 20b | Present results of all statistical syntheses conducted. If meta-analysis was done, present for each the summary estimate and its precision (e.g. confidence/credible interval) and measures of statistical heterogeneity. If comparing groups, describe the direction of the effect. | Results, page 16, Figure 2. |
|  | 20c | Present results of all investigations of possible causes of heterogeneity among study results. | Results, pages 15-22. |
|  | 20d | Present results of all sensitivity analyses conducted to assess the robustness of the synthesized results. |  |
| Reporting biases | 21 | Present assessments of risk of bias due to missing results (arising from reporting biases) for each synthesis assessed. |  |
| Certainty of evidence | 22 | Present assessments of certainty (or confidence) in the body of evidence for each outcome assessed. | Results, pages 15-22. |
| **DISCUSSION** | | |  |
| Discussion | 23a | Provide a general interpretation of the results in the context of other evidence. | Discussion page 22-23, paragraph 1 |
|  | 23b | Discuss any limitations of the evidence included in the review. | Discussion, strengths and limitations subheading, page 25-26. |
|  | 23c | Discuss any limitations of the review processes used. | Discussion, strengths and limitations subheading, page 25-26. |
|  | 23d | Discuss implications of the results for practice, policy, and future research. | Discussion, future research directions and clinical implications subheading, page 23-25. |
| **OTHER INFORMATION** | | |  |
| Registration and protocol | 24a | Provide registration information for the review, including register name and registration number, or state that the review was not registered. | Methods, page 5, paragraph 1 |
|  | 24b | Indicate where the review protocol can be accessed, or state that a protocol was not prepared. | Methods, page 5, paragraph 1 |
|  | 24c | Describe and explain any amendments to information provided at registration or in the protocol. |  |
| Support | 25 | Describe sources of financial or non-financial support for the review, and the role of the funders or sponsors in the review. | Page 27, funding source subheading. |
| Competing interests | 26 | Declare any competing interests of review authors. | Page 27, declaration of conflict of interest subheading. |
| Availability of data, code and other materials | 27 | Report which of the following are publicly available and where they can be found: template data collection forms; data extracted from included studies; data used for all analyses; analytic code; any other materials used in the review. | Page 27, data availability subheading. |

*From: Page MJ, McKenzie JE, Bossuyt PM, Boutron I, Hoffmann TC, Mulrow CD, et al. The PRISMA 2020 statement: an updated guideline for reporting systematic reviews. BMJ 2021;372:n71. doi: 10.1136/bmj.n71. This work is licensed under CC BY 4.0. To view a copy of this license, visit* [*https://creativecommons.org/licenses/by/4.0/*](https://url.avanan.click/v2/r02/___https://creativecommons.org/licenses/by/4.0/___.YXAxZTpjYW1icmlkZ2Vvcmc6YTpvOjliY2FkOWQ0ZWJkZjA0NTM5YjFkYjhiMjFmNTVmZDZlOjc6ZWM3NToxYjE4N2U0YWIwZTQ1Yzc3NjAyZWYzMjJkZTRhMTEwODUzYTJjY2Q0MmVhMGJiMzc0NDVjZjg0MTc4ZDFiNDU0OnA6VDpG)

**Table S2.** Full search strings used for each database

| **Database** | **Search String** |
| --- | --- |
| **PubMed** | ("auditory hallucination*" OR "hearing voices" OR "voice-hearing" OR "auditory hallucinations" OR ("voices" AND "psychosis"))  AND  ("Schizophrenia"[MeSH Terms] OR schizophren* OR "psychotic disorders" OR "psychosis" OR "schizoaffective disorder")  AND  ("Treatment Outcome"[MeSH Terms] OR "therapy" OR "treatment efficacy" OR "antipsychotic agents"[MeSH Terms] OR "Clozapine" OR "Haloperidol" OR "Olanzapine" OR "Risperidone" OR "Aripiprazole" OR "CBT" OR "Cognitive Behavioral Therapy" OR "cognitive remediation therapy" OR "psychoeducation" OR "family therapy" OR "supportive therapy" OR "electroconvulsive therapy" OR "rTMS" OR "transcranial magnetic stimulation" OR "tDCS" OR "transcranial direct current stimulation" OR "Deep Brain Stimulation" OR "DBS" OR "virtual reality therapy" OR "VR therapy" OR "avatar therapy" OR "digital intervention" OR "mHealth" OR "eHealth" OR "psychedelic-assisted psychotherapy" OR "psilocybin") |
| **Embase** | ("auditory hallucination*" OR "hearing voices" OR "voice-hearing" OR "hallucinations, auditory" OR ("voices" AND "psychosis"))  AND  ("schizophrenia" OR schizophren* OR "psychotic disorders" OR "psychosis" OR "schizoaffective disorder")  AND  ("treatment outcome" OR "treatment efficacy" OR "therapy" OR "antipsychotic agents" OR "Clozapine" OR "Haloperidol" OR "Olanzapine" OR "Risperidone" OR "Aripiprazole" OR "Cognitive Behavioral Therapy" OR "CBT" OR "cognitive remediation therapy" OR "psychoeducation" OR "family therapy" OR "supportive therapy" OR "electroconvulsive therapy" OR "rTMS" OR "transcranial magnetic stimulation" OR "tDCS" OR "transcranial direct current stimulation" OR "Deep Brain Stimulation" OR "DBS" OR "virtual reality therapy" OR "avatar therapy" OR "digital intervention" OR "mHealth" OR "eHealth" OR "psychedelic-assisted psychotherapy" OR "psilocybin") |
| **PsycInfo** | ("auditory hallucination*" OR "hearing voices" OR "voice-hearing" OR "hallucinations, auditory" OR ("voices" AND "psychosis"))  AND  ("schizophrenia" OR schizophren* OR "psychotic disorders" OR "psychosis" OR "schizoaffective disorder")  AND  ("treatment outcome" OR "treatment efficacy" OR "therapy" OR "antipsychotic agents" OR "Clozapine" OR "Haloperidol" OR "Olanzapine" OR "Risperidone" OR "Aripiprazole" OR "Cognitive Behavioral Therapy" OR "CBT" OR "cognitive remediation therapy" OR "psychoeducation" OR "family therapy" OR "supportive therapy" OR "electroconvulsive therapy" OR "rTMS" OR "transcranial magnetic stimulation" OR "tDCS" OR "transcranial direct current stimulation" OR "Deep Brain Stimulation" OR "DBS" OR "virtual reality therapy" OR "avatar therapy" OR "digital intervention" OR "mHealth" OR "eHealth" OR "psychedelic-assisted psychotherapy" OR "psilocybin") |
| **Medline** | exp "Auditory Hallucinations"/ OR "auditory hallucination".tw OR "hearing voices"/OR "voice-hearing".tw OR ("voices" AND "psychosis").tw  AND  (exp "Schizophrenia"/ OR schizophren*.tw OR exp "Psychotic Disorders"/ OR "psychosis" / OR "schizoaffective disorder".tw)  AND  (exp "Treatment Outcome"/ OR "treatment efficacy".tw OR "therapy".tw OR exp "Antipsychotic Agents"/ OR "Clozapine".tw OR "Haloperidol".tw OR "Olanzapine".tw OR "Risperidone".tw OR "Aripiprazole".tw OR "Cognitive Behavioral Therapy".tw OR "CBT".tw OR "cognitive remediation therapy".tw OR "psychoeducation".tw OR "family therapy".tw OR "supportive therapy".tw OR exp "Electroconvulsive Therapy"/ OR "rTMS".tw OR "transcranial magnetic stimulation".tw OR "tDCS".tw OR "transcranial direct current stimulation".tw OR "Deep Brain Stimulation".tw OR "DBS".tw OR "virtual reality therapy".tw OR "avatar therapy".tw OR "digital intervention".tw OR "mHealth".tw OR "eHealth".tw OR "psychedelic-assisted psychotherapy".tw OR "psilocybin".tw) |
| **Web Of Science** | ("auditory hallucination*" OR "hearing voices" OR "voice-hearing" OR "hallucinations, auditory" OR ("voices" AND "psychosis"))  AND  ("schizophrenia" OR schizophren* OR "psychotic disorders" OR "psychosis" OR "schizoaffective disorder")  AND  ("treatment outcome" OR "treatment efficacy" OR "therapy" OR "antipsychotic agents" OR "Clozapine" OR "Haloperidol" OR "Olanzapine" OR "Risperidone" OR "Aripiprazole" OR "Cognitive Behavioral Therapy" OR "CBT" OR "cognitive remediation therapy" OR "psychoeducation" OR "family therapy" OR "supportive therapy" OR "electroconvulsive therapy" OR "rTMS" OR "transcranial magnetic stimulation" OR "tDCS" OR "transcranial direct current stimulation" OR "Deep Brain Stimulation" OR "DBS" OR "virtual reality therapy" OR "avatar therapy" OR "digital intervention" OR "mHealth" OR "eHealth" OR "psychedelic-assisted psychotherapy" OR "psilocybin") |


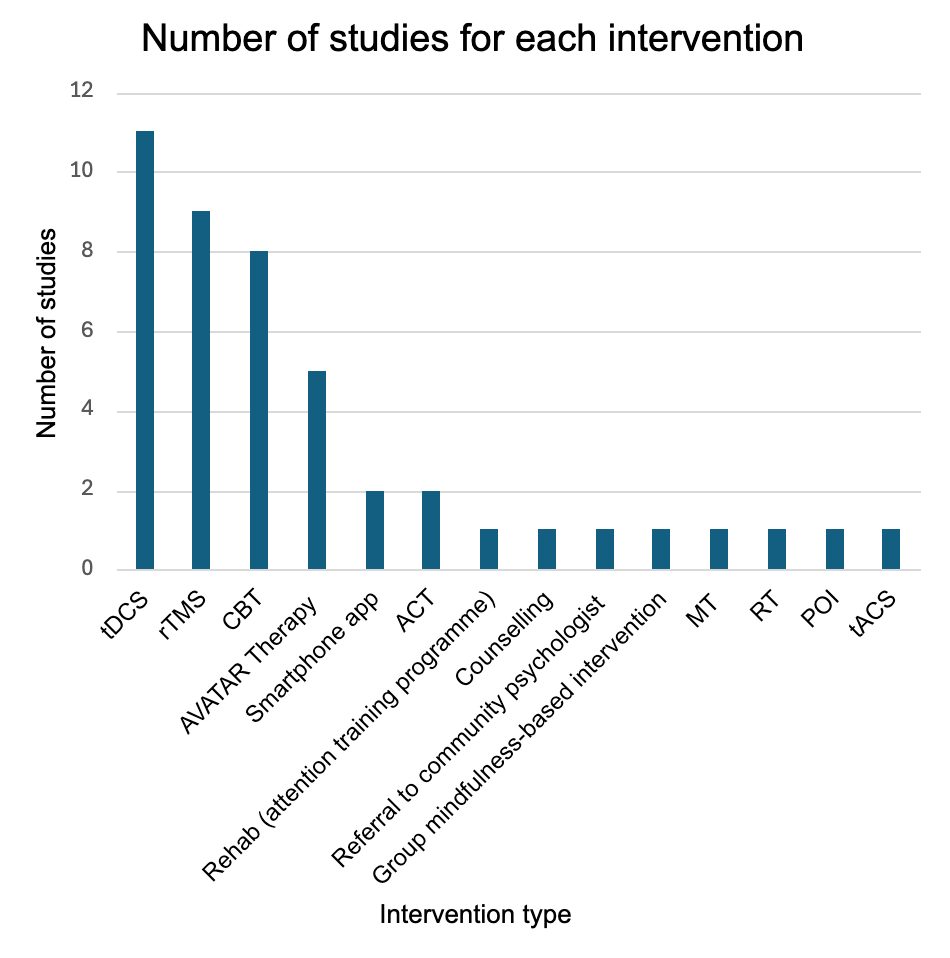


**Fig. S1.** Quantity of each treatment type in the meta-analysis.

1. **List of all included papers:**
2. Fitzgerald PB, McQueen S, Daskalakis ZJ, Hoy KE. A negative pilot study of daily bimodal transcranial direct current stimulation in schizophrenia. Brain Stimul. 2014;7(6):813-6.
3. Klirova M, Horacek J, Novak T, Cermak J, Spaniel F, Skrdlantova L, Mohr P, Höschl C. Individualized rTMS neuronavigated according to regional brain metabolism (18 FGD PET) has better treatment effects on auditory hallucinations than standard positioning of rTMS: a double-blind, sham-controlled study. Eur Arch Psychiatry Clin Neurosci. 2013;263:475-84.
4. Krakvik B, Grawe RW, Hagen R, Stiles TC. Cognitive behaviour therapy for psychotic symptoms: a randomized controlled effectiveness trial. Behav Cogn Psychother. 2013;41(5):511-24.
5. Bais L, Vercammen A, Stewart R, van Es F, Visser B, Aleman A, et al. Short and long term effects of left and bilateral repetitive transcranial magnetic stimulation in schizophrenia patients with auditory verbal hallucinations: a randomized controlled trial. PLoS One. 2014;9(10):e108828.
6. Freeman D, Waite F, Startup H, Myers E, Lister R, McInerney J, et al. Efficacy of cognitive behavioural therapy for sleep improvement in patients with persistent delusions and hallucinations (BEST): a prospective, assessor-blind, randomised controlled pilot trial. Lancet Psychiatry. 2015;2(11):975-83.
7. Naeem F, Saeed S, Irfan M, Kiran T, Mehmood N, Gul M, et al. Brief culturally adapted CBT for psychosis (CaCBTp): A randomized controlled trial from a low income country. Schizophr Res. 2015;164(1-3):143-8.
8. Ray P, Sinha VK, Tikka SK. Adjuvant low-frequency rTMS in treating auditory hallucinations in recent-onset schizophrenia: A randomized controlled study investigating the effect of high-frequency priming stimulation. Ann Gen Psychiatry. 2015;14.
9. Chadwick P, Strauss C, Jones AM, Kingdon D, Ellett L, Dannahy L, et al. Group mindfulness-based intervention for distressing voices: A pragmatic randomised controlled trial. Schizophr Res. 2016;175(1-3):168-73.
10. Frohlich F, Burrello TN, Mellin JM, Cordle AL, Lustenberger CM, Gilmore JH, et al. Exploratory study of once-daily transcranial direct current stimulation (tDCS) as a treatment for auditory hallucinations in schizophrenia. Eur Psychiatry. 2016;33:54-60.
11. Kimura H, Kanahara N, Takase M, Yoshida T, Watanabe H, Iyo M. A randomized, sham-controlled study of high frequency rTMS for auditory hallucination in schizophrenia. Psychiatry Res. 2016;241:190-4.
12. Koops S, van Dellen E, Schutte MJ, Nieuwdorp W, Neggers SF, Sommer IE. Theta Burst Transcranial Magnetic Stimulation for Auditory Verbal Hallucinations: Negative Findings From a Double-Blind-Randomized Trial. Schizophr Bull. 2016;42(1):250-7.
13. Lopez-Luengo B, Muela-Martinez JA. Preliminary study of a rehabilitation program based on attentional processes to treat auditory hallucinations. Cogn Neuropsychiatry. 2016;21(4):315-34.
14. Mondino M, Jardri R, Suaud-Chagny M-F, Saoud M, Poulet E, Brunelin J. Effects of Fronto-Temporal Transcranial Direct Current Stimulation on Auditory Verbal Hallucinations and Resting-State Functional Connectivity of the Left Temporo-Parietal Junction in Patients With Schizophrenia. Schizophr Bull. 2016;42(2):318-26.
15. Naeem F, Johal R, McKenna C, Rathod S, Ayub M, Lecomte T, et al. Cognitive Behavior Therapy for psychosis based Guided Self-help (CBTp-GSH) delivered by frontline mental health professionals: Results of a feasibility study. Schizophr Res. 2016;173(1-2):69-74.
16. Gottlieb JD, Gidugu V, Maru M, Tepper MC, Davis MJ, Greenwold J, et al. Randomized controlled trial of an internet cognitive behavioral skills-based program for auditory hallucinations in persons with psychosis. Psychiatr Rehabil J. 2017;40(3):283-92.
17. Hayward M, Jones A-M, Bogen-Johnston L, Thomas N, Strauss C. Relating Therapy for distressing auditory hallucinations: A pilot randomized controlled trial. Schizophr Res. 2017;183:137-42.
18. Husain MO, Chaudhry IB, Mehmood N, Rehman RU, Kazmi A, Hamirani M, et al. Pilot randomised controlled trial of culturally adapted cognitive behavior therapy for psychosis (CaCBTp) in Pakistan. BMC Health Serv Res. 2017;17(1):808.
19. Paillère‐Martinot ML, Galinowski A, Plaze M, Andoh J, Bartrés‐Faz D, Bellivier F, et al. Active and placebo transcranial magnetic stimulation effects on external and internal auditory hallucinations of schizophrenia. Acta Psychiatr Scand. 2017;135(3):228-38.
20. Schnackenberg J, Fleming M, Martin CR. A randomised controlled pilot study of Experience Focused Counselling with voice hearers. Psychosis. 2017;9(1):12-24.
21. Shawyer F, Farhall J, Thomas N, Hayes SC, Gallop R, Copolov D, Castle DJ. Acceptance and commitment therapy for psychosis: randomised controlled trial. Br J Psychiatry. 2017;210(2):140-8.
22. Bose A, Shivakumar V, Agarwal SM, Kalmady SV, Shenoy S, Sreeraj VS, et al. Efficacy of fronto-temporal transcranial direct current stimulation for refractory auditory verbal hallucinations in schizophrenia: A randomized, double-blind, sham-controlled study. Schizophr Res. 2018;195:475-80.
23. Chang C-C, Tzeng N-S, Chao C-Y, Yeh C-B, Chang H-A. The Effects of Add-on Fronto-Temporal Transcranial Direct Current Stimulation (tDCS) on Auditory Verbal Hallucinations, Other Psychopathological Symptoms, and Insight in Schizophrenia: A Randomized, Double-Blind, Sham-Controlled Trial. Int J Neuropsychopharmacol. 2018;21(11):979-87.
24. Craig TK, Rus-Calafell M, Ward T, Leff JP, Huckvale M, Howarth E, et al. AVATAR therapy for auditory verbal hallucinations in people with psychosis: a single-blind, randomised controlled trial. Lancet Psychiatry. 2018;5(1):31-40.
25. Hazell CM, Hayward M, Cavanagh K, Jones A-M, Strauss C. Guided self-help cognitive-behaviour Intervention for VoicEs (GiVE): Results from a pilot randomised controlled trial in a transdiagnostic sample. Schizophr Res. 2018;195:441-7.
26. Koops S, Blom JD, Bouachmir O, Slot MI, Neggers B, Sommer IE. Treating auditory hallucinations with transcranial direct current stimulation in a double-blind, randomized trial. Schizophr Res. 2018;201:329-36.
27. Mellin JM, Alagapan S, Lustenberger C, Lugo CE, Alexander ML, Gilmore JH, et al. Randomized trial of transcranial alternating current stimulation for treatment of auditory hallucinations in schizophrenia. Eur Psychiatry. 2018;51:25-33.
28. Percie du Sert O, Potvin S, Lipp O, Dellazizzo L, Laurelli M, Breton R, et al. Virtual reality therapy for refractory auditory verbal hallucinations in schizophrenia: A pilot clinical trial. Schizophr Res. 2018;197:176-81.
29. Ertekin Pinar S Rn P, Tel H Rn P. The Effect of Music on Auditory Hallucination and Quality of Life in Schizophrenic Patients: A Randomised Controlled Trial. Issues Ment Health Nurs. 2019;40(1):50-7.
30. Kantrowitz JT, Sehatpour P, Avissar M, Horga G, Gwak A, Hoptman MJ, et al. Significant improvement in treatment resistant auditory verbal hallucinations after 5 days of double-blind, randomized, sham controlled, fronto-temporal, transcranial direct current stimulation (tDCS): A replication/extension study. Brain Stimul. 2019;12(4):981-91.
31. Lindenmayer JP, Kulsa MKC, Sultana T, Kaur A, Yang R, Ljuri I, et al. Transcranial direct-current stimulation in ultra-treatment-resistant schizophrenia. Brain Stimul. 2019;12(1):54-61.
32. Bell IH, Rossell SL, Farhall J, Hayward M, Lim MH, Fielding-Smith SF, et al. Pilot randomised controlled trial of a brief coping-focused intervention for hearing voices blended with smartphone-based ecological momentary assessment and intervention (SAVVy): Feasibility, acceptability and preliminary clinical outcomes. Schizophr Res. 2020;216:479-87.
33. Lüdtke T, Platow-Kohlschein H, Rüegg N, Berger T, Moritz S, Westermann S. Mindfulness mediates the effect of a psychological online intervention for psychosis on self-reported hallucinations: A secondary analysis of voice hearers from the EviBaS trial. Front Psychiatry. 2020;11.
34. Mortan Sevi O, Tekinsav Sutcu S, Yesilyurt S, Turan Eroglu S, Gunes B. Comparison of the Effectiveness of Two Cognitive-Behavioral Group Therapy Programs for Schizophrenia: Results of a Short-Term Randomized Control Trial. Community Ment Health J. 2020;56(2):222-8.
35. Valiengo LdCL, Goerigk S, Gordon PC, Padberg F, Serpa MH, Koebe S, et al. Efficacy and safety of transcranial direct current stimulation for treating negative symptoms in schizophrenia: A randomized clinical trial. JAMA Psychiatry. 2020;77(2):121-9.
36. Dellazizzo L, Potvin S, Phraxayavong K, Dumais A. One-year randomized trial comparing virtual reality-assisted therapy to cognitive-behavioral therapy for patients with treatment-resistant schizophrenia. NPJ Schizophrenia. 2021;7(1).
37. El Ashry AM, Abd El Dayem SM, Ramadan FH. Effect of applying “acceptance and commitment therapy” on auditory hallucinations among patients with schizophrenia. Arch of Psychriatr Nurs. 2021;35(2):141-52.
38. Klein HS, Vanneste S, Pinkham AE. The limited effect of neural stimulation on visual attention and social cognition in individuals with schizophrenia. Neuropsychologia. 2021;157.
39. Liang N, Li X, Guo X, Liu S, Liu Y, Zhao W, et al. Visual P300 as a neurophysiological correlate of symptomatic improvement by a virtual reality-based computer AT system in patients with auditory verbal hallucinations: A Pilot study. J Psychiatr Res. 2022;151:261-71.
40. Solar A, Bennett K, Hulse G. Clinical psychology referral for individuals with auditory verbal hallucinations and schizophrenia: Therapy engagement, hallucination severity and distress. Australas Psychiatry. 2022;30(4):452-7.
41. Tyagi P, Dhyani M, Khattri S, Tejan V, Tikka SK, Garg S. “Efficacy of intensive bilateral Temporo-Parietal Continuous theta-burst Stimulation for Auditory VErbal hallucinations (TPC-SAVE) in schizophrenia: A randomized sham-controlled trial”. Asian J Psychiatry. 2022;74:103176.
42. Zhang M, Force RB, Walker C, Ahn S, Jarskog LF, Frohlich F. Alpha transcranial alternating current stimulation reduces depressive symptoms in people with schizophrenia and auditory hallucinations: a double-blind, randomized pilot clinical trial. Nat Schizophrenia. 2022;8(1):114.
43. Xie Y, Guan M, He Y, Wang Z, Ma Z, Fang P, et al. The Static and dynamic functional connectivity characteristics of the left temporoparietal junction region in schizophrenia patients with auditory verbal hallucinations during low-frequency rTMS treatment. Front Psychiatry. 2023;14:1071769.
44. Garety PA, Edwards CJ, Jafari H, Emsley R, Huckvale M, Rus-Calafell M, Fornells-Ambrojo M, Gumley A, Haddock G, Bucci S, McLeod HJ. Digital AVATAR therapy for distressing voices in psychosis: the phase 2/3 AVATAR2 trial. Nat Med. 2024;28:1-1.
45. Hua Q, Wang L, He K, Sun J, Xu W, Zhang L, et al. Repetitive Transcranial Magnetic Stimulation for Auditory Verbal Hallucinations in Schizophrenia: A Randomized Clinical Trial. JAMA Netw Open. 2024;7(11):e2444215.
46. Jongeneel A, Delespaul P, Tromp N, Scheffers D, van der Vleugel B, de Bont P, Kikkert M, Croes CF, Staring AB, Riper H, van der Gaag M. Effects on voice hearing distress and social functioning of unguided application of a smartphone app—a randomized controlled trial. Internet Interv. 2024;35:100717.


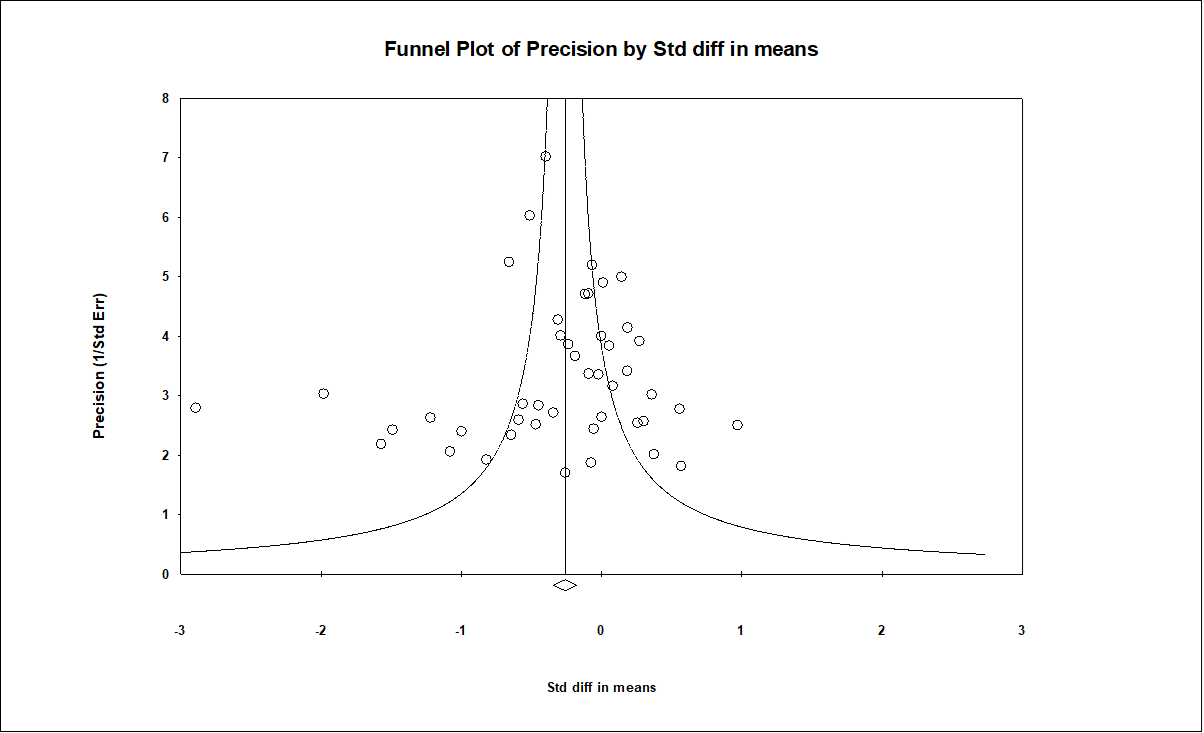


**Fig. S2.** Funnel plot of precision for publication bias.

**Table S3**. *QualSyst* bias scores for each study.

|  |  |
| --- | --- |
| Study | **QualSyst Bias Score** |
| Klirova M. et al. 2013 | 0.93 |
| Kråkvik B. et al. 2013 | 0.82 |
| Bais L. et al. 2014 | 0.93 |
| Fitzgerald P. et al. 2014 | 0.93 |
| Freeman D. et al. 2015 | 0.93 |
| Naeem F. et al. 2015 | 0.83 |
| Ray P. et al. 2015 | 0.93 |
| Fröhlich F. et al. 2016 | 0.93 |
| Kimura H. et al. 2016 | 0.86 |
| Koops S. et al. 2016 | 0.93 |
| López-Luengo B. et al. 2016 | 0.86 |
| Mondino M. et al. 2016 | 0.91 |
| Naeem F. et al. 2016 | 0.89 |
| Chadwick P. et al. 2016 | 0.89 |
| Gottlieb J. et al. 2017 | 0.83 |
| Hayward M. et al. 2017 | 0.86 |
| Husain M. et al. 2017 | 0.82 |
| Paillere-Martinot M. et al. 2017 | 0.93 |
| Schnackenberg J. et al. 2017 | 0.72 |
| Shawyer F. et al. 2017 | 0.96 |
| Bose A. et al. 2018 | 0.96 |
| Chang C. et al. 2018 | 0.93 |
| Craig T. et al. 2018 | 0.89 |
| Hazell C. et al. 2018 | 0.82 |
| Koops S. et al. 2018 | 0.93 |
| Mellin J. et al. 2018 | 0.93 |
| Percie du Sert O. et al. 201852 | 0.86 |
| Pinar S. et al. 2018 | 0.70 |
| Kantrowitz J. et al. 201954 | 0.93 |
| Lindenmayer J. et al. 201955 | 0.93 |
| Bell I. et al. 2020 | 0.86 |
| Lüdtke T. et al. 2020 | 0.79 |
| Sevi O. et al. 2020 | 0.86 |
| Valiengo L. et al.2020 | 0.96 |
| Dellazizzo L. et al. 2021 | 0.82 |
| El Ashry A. et al. 2021 | 0.76 |
| Klein H. et al. 2021 | 0.89 |
| Liang N. et al. 2022 | 0.86 |
| Solar A. et al. 2022 | 0.93 |
| Tyagi P. et al. 2022 | 0.93 |
| Zhang M. et al. 2022 | 0.93 |
| Xie Y. et al. 2023 | 0.89 |
| Garety P. et al. 2024 | 0.86 |
| Hua Q. et al. 2024 | 0.96 |
| Jongeneel A. et al 2024 | 0.89 |
